# Visual Disturbance During Pregnancy: A Case Report of Central Serous Chorioretinopathy

**DOI:** 10.7759/cureus.94473

**Published:** 2025-10-13

**Authors:** Tejaswi Mandadi, Sriram Simakurthy

**Affiliations:** 1 Department of Ophthalmology, Sankara Eye Hospital, Hyderabad, IND; 2 Department of Vitreo Retinal Services, Sankara Eye Hospital, Hyderabad, IND

**Keywords:** central serous chorioretinopathy, pregnancy, pregnancy complications, retinal pigment epithelial dysfunction, spontaneous resolution, subretinal fluid

## Abstract

Central serous chorioretinopathy (CSCR) is defined as a fluid-filled detachment of the neurosensory retina due to focal leakage at the retinal pigment epithelium. Although CSCR is more common in men, it has a strong association with pregnancy, particularly in the later trimesters. We report the case of a 34-year-old woman at 32 weeks of gestation who presented with unilateral CSCR. The clinical manifestations included a central scotoma without pain or redness. Fundus examination and optical coherence tomography confirmed the diagnosis. The patient was managed conservatively with reassurance and lifestyle modifications. At five weeks postpartum, complete resolution of the subretinal fluid was documented without any intervention. Pregnancy-associated CSCR is linked to elevated cortisol levels and hemodynamic changes. It usually follows a benign course with spontaneous resolution postpartum, although chronic or recurrent disease can occur. Diagnostic modalities are limited by safety concerns, and treatment is generally avoided unless complications develop. This case highlights that CSCR can occur during pregnancy and is typically self-limiting. Educating pregnant women about possible visual disturbances and ensuring close follow-up are essential.

## Introduction

Pregnancy induces several changes in the body of the mother to meet the increased metabolic demands of the fetus, such as a hypercoagulable state to reduce the risk of excessive blood loss during delivery and immunologic adjustments to protect both the mother and the fetus. Pregnancy also brings about physiological alterations in the eye, which may exacerbate preexisting ocular diseases or lead to new manifestations. One such condition is central serous chorioretinopathy (CSCR) [1]. Although commonly seen in middle-aged men and women, it is of rare occurrence in pregnant women.

Other ocular conditions associated with pregnancy include retinal vein occlusion, retinal artery occlusion, diabetic retinopathy, and hypertensive-related retinopathy. The latter may present in association with systemic complications of pregnancy, including preeclampsia, eclampsia, and hemolysis, elevated liver enzymes, and low platelet (HELLP) count syndrome [1].

## Case presentation

A 34-year-old woman, at 32 weeks of gestation (primigravida), presented to the outpatient department with a sudden-onset central scotoma in the right eye (RE) for the past five days. There was no history of ocular pain, redness, floaters, flashes, or trauma. Her past ocular history was unremarkable. At presentation, her blood pressure was 130/84 mmHg. There was no history of gestational diabetes, preeclampsia, or systemic steroid usage.

**Figure 1:**
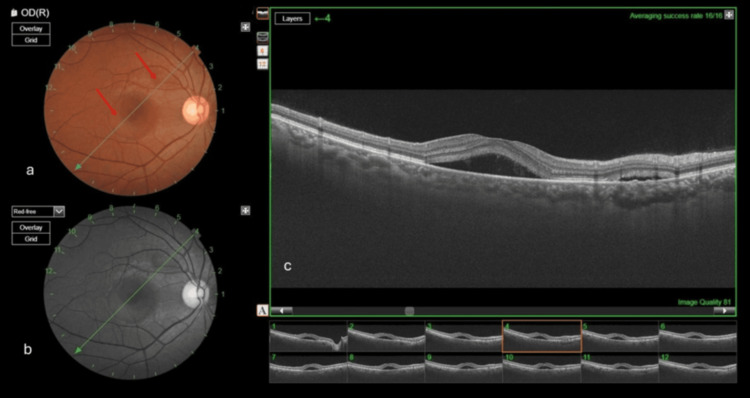
RE retina scan (a) Fundus color photograph of the RE showing two distinct neurosensory detachments (red arrows). (b) Red-free image of the RE highlighting subretinal fluid at the macula. (c) Swept-source OCT of the RE showing fluid collection above the RPE RE: right eye; RPE: retinal pigment epithelium; OCT: optical coherence tomography

On examination, the best corrected visual acuity was 6/6 in both eyes. The anterior segment and pupillary reactions were normal, and intraocular pressure measurements were 18 mmHg in the RE and 17 mmHg in the left eye (LE). Dilated fundus examination of the RE revealed a clear media, a normal optic disk, and an absent foveal reflex with subretinal fluid collection at the macula. The LE fundus was within normal limits. Optical coherence tomography (OCT) of the RE demonstrated two distinct neurosensory detachments (Figure 1), confirming the diagnosis of unilateral CSCR associated with pregnancy.

**Figure 2:**
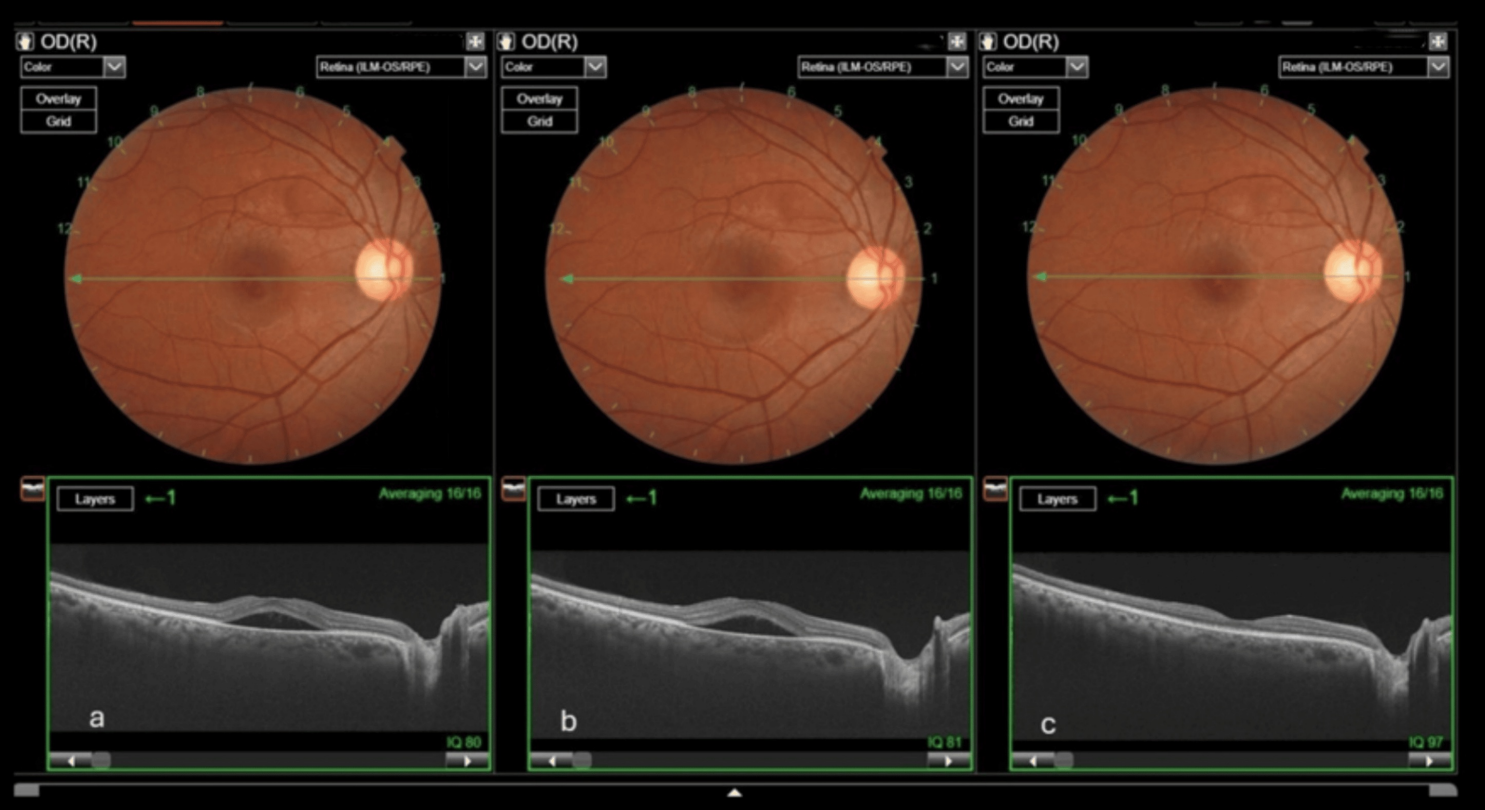
Serial photographs showing subretinal fluid and the course of CSCR (a) At presentation (32 weeks of gestational age). (b) Remained stable without progression at the first follow-up (35 weeks of gestational age). (c) At the second follow-up (five weeks postpartum), which shows complete resolution of subretinal fluid CSCR: central serous chorioretinopathy

The patient was informed about her clinical condition, reassured about postpartum recovery, and advised on general lifestyle modifications, including stress reduction. The obstetrician was also updated about her ocular condition. At the first follow-up, at 35 weeks of gestation, clinical and OCT findings of the RE remained stable without progression. At the second follow-up, five weeks postpartum, complete resolution of subretinal fluid was observed on both fundus examination and OCT (Figure 2).

## Discussion

CSCR is reported to be 10 times more common in men, but pregnancy is a risk factor in women. CSCR is characterized by elevated endogenous cortisol and catecholamines, which increase the permeability of the blood-retinal barrier and cause secondary RPE dysfunction [2]. In addition, the increased blood volume and hemodynamic changes during pregnancy contribute to the development of CSCR. Accumulation of subretinal fluid leads to neurosensory retinal detachment in the macula at the level of the RPE.

Pregnancy-associated CSCR (pCSCR) may present with distinctive features such as white subretinal exudates. One study reported these exudates in 90% of pCSCR cases compared to 20% in nonpregnancy cases [3]. Quillen et al. [4] further noted that such exudates were more common in patients receiving corticosteroid therapy. The absence of retinal exudates in our patient could therefore be attributed to the lack of corticosteroid exposure before or during pregnancy. Several risk factors that predispose pregnant women to CSCR include glucocorticoid use, Cushing syndrome, obstructive sleep apnea, preeclampsia, hypertension, and emotional stress [5].

Diagnostic tests such as fluorescein angiography (FA) and OCT are performed to rule out other diagnoses and guide treatment. OCT is the preferred modality during pregnancy, as it is noninvasive and safe, revealing findings such as small pigment epithelial detachments and hyperreflective fibrinous subretinal fluid [6]. In contrast, FA, which typically demonstrates patterns such as an inkblot (31%), a smokestack (12%), or a minimally enlarging spot (7%), is usually avoided during pregnancy due to potential risks.

The natural course of CSCR during pregnancy is usually benign, with spontaneous resolution of subretinal fluid within one to three months after delivery. A decrease in visual acuity is insignificant; however, patients may report a reduction in vision quality. In rare cases, chronic CSCR can develop, persisting for over six months and leading to irreversible photoreceptor damage, subretinal fibrosis, or macular scarring [7,8]. Choroidal neovascularization, although less common, occurs in approximately 8% of cases and can significantly impair vision [9].

Treatment options for CSCR include observation, photodynamic therapy, argon laser photocoagulation, subthreshold micropulse laser therapy, anti-VEGF injections, and systemic agents such as carbonic anhydrase inhibitors and anticorticosteroid therapies. Most of these are avoided during pregnancy due to potential risks to fetal development. Subthreshold micropulse laser has recently shown promising results in chronic CSCR and is considered the safest therapeutic option during pregnancy, as it carries no systemic risk [10]. In our patient, the clinical features were highly consistent with pCSCR. She presented in the third trimester, aligning with the typical period of occurrence. Fundus examination revealed a unilateral serous macular detachment without signs of intraocular inflammation. Symptoms resolved spontaneously postpartum, with restoration of normal vision, which is the expected course of the disease. No active intervention was performed in this case, as indications such as persistence beyond four months, recurrence in an eye with prior visual deficit, or bilateral involvement were absent.

Differential diagnoses include exudative retinal detachment secondary to preeclampsia. However, this was unlikely in our patient due to the absence of hypertension, proteinuria, and pedal edema.

## Conclusions

This case highlights that CSCR can occur during pregnancy and is commonly self-limiting. Conservative management and reassurance should be the first-line management for CSCR in pregnant women. This case underscores the importance of educating pregnant women about the possibility of visual disturbances during gestation. Close follow-up is essential until complete resolution, with timely intervention reserved for cases at risk of irreversible visual loss. For safety reasons, invasive diagnostic and therapeutic procedures are generally avoided during pregnancy, which limits our understanding of the underlying pathophysiology and treatment outcomes in this group.
